# Inflammation driven by NF-ĸB compromises bone formation by inducing the differentiation of osteoblasts into atypical inflammatory osteocyte-like cells

**DOI:** 10.1038/s41413-026-00546-0

**Published:** 2026-08-04

**Authors:** Gaurav Swarnkar, Md Fahim Ahmad, Marwa Zeyad, Abdul Malik Tyagi, Musarrat Naaz, Gabriel Mbalaviele, Yousef Abu-Amer

**Affiliations:** 1 Department of Orthopedic Surgery, Washington University School of Medicine, St. Louis, MO USA; 2 Department of Cell Biology & Physiology, Washington University School of Medicine, St. Louis, MO USA; 3 Divison of Bone and Mineral Research, Washington University School of Medicine, St. Louis, MO USA; 4 Shriners Hospital for Children, St. Louis, MO USA

**Keywords:** Bone, Pathogenesis

## Abstract

Inflammation causes bone loss by dysregulating the differentiation and functions of osteoclasts, osteoblasts, and osteocytes. This process can be modeled by the expression of constitutively activated IKK2 (IKK2ca), a strategy that we leveraged to investigate the mechanisms through which inflammation negatively affects cells of the osteoblast/osteocyte lineage. We found that mice expressing IKK2ca in osteoblasts exhibit significant bone loss. Mechanistically, IKK2ca downregulates the expression of osteoblast genes while inducing the differentiation of bone-forming osteoblasts into catabolic osteocytes like cells, expressing high levels of *Podoplanin*, *Fgf23*, *Dkk1* and *Sclerostin*. We term these atypical inflammatory osteocyte-like cells (aiOCy-L cells) as they highly express inflammatory and senescence markers and promote osteoclast differentiation as well. Additional data show that inflammation induces abnormal differentiation of OB into aiOCy-L cells through upregulating mTOR and glycolysis. In summary, we uncovered a mechanism by which inflammation alters osteoblast differentiation and fate decision via the IKK2/mTOR/glycolysis axis.

## Introduction

Inflammatory responses, if not resolved in a timely manner, contribute to the pathogenesis of skeletal diseases. Therefore, deciphering the molecular mechanism(s) underlying inflammation-mediated osteopenia will contribute to long-term management of bone health in individuals with underlying inflammatory diseases. Osteoblasts (OBs) are the principal bone-forming cells, and their steady activity is crucial to maintain bone homeostasis. Various reports examining the role of inflammation suggest that inflammation hinders bone formation by inhibiting OB differentiation and function while promoting apoptosis. In parallel, inflammatory factors (e.g., TNFα and IL17) promote Rankl secretion, thereby boosting osteoclast (OC) differentiation and function.^1–4^ Mechanistically, it has been shown that inflammatory signals, such as TNFα mediated-activation of canonical NF-ĸB signaling interfere with SMAD complex in OBs leading to inhibition of Runx2 transcription.^1,5,6^ In other studies, inflammatory signals were shown to inhibit OB function by targeting MAPK pathways, IGF1 signaling, transcription factors like Runx2, Smurf1/2, JAK/STAT signaling, WNT signaling or activating NF-κB.^7^

OBs differentiate into osteocytes (OCy) which constitute over 90% of total cells in bone. OCy are actively involved in bone remodeling by expressing and releasing proteins that signal to OBs, OCs, and other bone-residing cells.^8,9^ Traditionally, OCy are considered terminally mature OBs that have been trapped within the mineralized bone matrix, express canalicular networks that sense fluctuations in their cellular niche environment, and respond accordingly under physiologic and pathologic strains to modulate bone homeostasis.^8,10–14^ While the OCy secreted factors that modulate OB and OC functions have been elucidated, the mechanisms that govern OCy dysfunction during skeletal inflammation are less clear.^15^ Several studies that were performed using inflammatory mediators (such as TNFα and IL1β) have shown induction of OCy apoptosis and release of cytokines that contribute towards OC-mediated inflammatory bone loss.^16–20^ However, these studies have been performed using non-physiological concentration of a few specific inflammatory signals and do not provide any significant mechanistic insights in response to the presence of multitude of inflammatory signals in disease conditions.

Numerous inflammatory and osteoclastogenic mediators including TNFα, IL1β, IL6, IL17, IL11, and RANKL have been implicated in bone catabolic responses, rendering investigation for associated molecular mechanism for management and therapeutic intervention challenging and imprecise. Notably, most of these factors converge at and require activation of the NF-κB pathway for their function.^21–25^ Consistently, it has also been established that members of the NF-κB pathway regulate physiological and pathological responses in the skeleton and extensively crosstalk with other metabolic systems.^25–27^ These facts position NF-κB and downstream signaling as an attractive well-defined target for molecular intervention and lessen the need to target individual cytokines. Therefore, to specifically identify the molecular mechanism associated with inflammation-induced dysregulation of OB function, we genetically activated inflammation in OBs by expressing constitutively active IKK2,^28^ thereafter referred to as IKK2ca.

Our findings demonstrate that the constitutive activation of IKK2, which mimics inflammation, promotes abnormal differentiation of OBs into an atypical inflammatory OCy-like cell (aiOCy-L cells). Specifically, IKK2ca regulates transcription of a subset of inflammatory, senescence, osteoclastogenic and anti-osteoblastogenic cytokines including *Il1β*, *Il6*, *Dkk1*, *Sost* and *Rankl* in OBs and induces atypical differentiation of OBs to catabolic aiOCy-L cells. Mechanistically, we further identified that IKK2ca regulates differentiation of OB to aiOCy-L cells by altering inflammation-induced energy metabolism evident by abnormally elevated mTOR and glycolysis. These findings highlight a novel metabolism-inflammatory crosstalk mechanism governing dysregulation of OBs and transition towards catabolic aiOCy-L phenotype during inflammation. Collectively, these findings offer a specific and defined road map to mitigate inflammation-induced bone loss.

## Results

### Constitutive activation of IKK2 in OBs, which mimics persistent inflammation, decreases bone mass

**Fig. 1:**
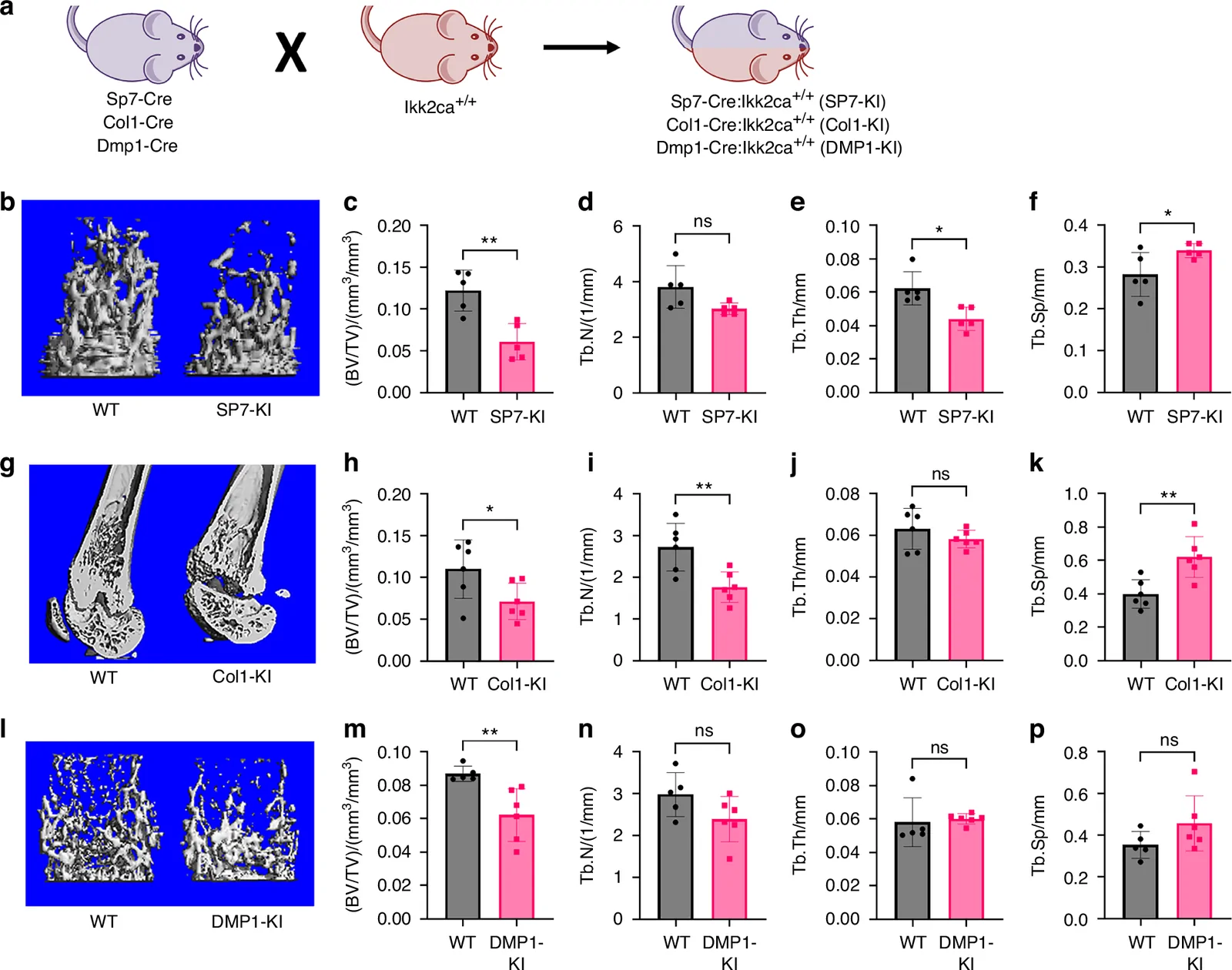
Constitutive activation of IKK2 in OBs, which mimics inflammation, decreases bone mass. **a** *Sp7*-cre, *Col1cre*-cre and *Dmp1*-cre mice were bred with *IKK2ca floxed* mice to generate *Sp7-Cre:IKK2ca*^*+/+*^ (SP7-KI), *Col1-Cre:IKK2ca*^*+/+*^ (Col1-KI) and *Dmp1-Cre:IKK2ca*^*+/+*^ (DMP1-KI) animals. 6–8 weeks old male and female animals were sacrificed, and long bones (femur) were used for microCT analysis. MicroCT analysis showing reduce bone mass in IKK2ca expressing mice under OB specific cre, **b**–**f** *Sp7-Cre*^*+/−*^*:IKK2ca*^*+/+*^ (SP7-KI), **g**–**k** *Col1-Cre*^*+/−*^*:IKK2ca*^*+/+*^ (Col1-KI), **l**–**p** *Dmp1-Cre:IKK2ca*^*+/+*^ (DMP1-KI) animals. BV/TV (Bone volume/Total volume), Tb.N (Trabecular number), Tb.Th (Trabecular thickness), Tb.Sp (Trabecular Separation). *n* = 5–6 per group. Data show mean ± S.D. *P < 0.05, **P < 0.01 (Student *t* test)

The mechanisms by which inflammation impairs OB differentiation and function are not well-defined owing to redundancy of pro-inflammatory cytokines. However, most of them converge at and require activation of the NF-κB pathway for their function. Therefore, we used a constitutive active genetic IKK2ca expression model to activate NF-κB, mimicking persistent inflammation (Supplementary Data 1). To specifically examine the role of inflammation and NF-ĸB activation on OB differentiation and function, we conditionally activated NF-ĸB in OB cells, by breeding OB specific cre mice (*Sp7-Cre and Col1-Cre)* and late OB and OCy specific cre (*Dmp1-cre)* with *IKK2ca−*floxed mice to generate *Sp7-Cre:IKK2ca*^*+/+*^ knock-in (SP7-KI), *Col1-Cre:IKK2ca*^*+/+*^ knock-in (Col1-KI) and *Dmp1-Cre:IKK2ca*^*+/+*^ knock-in (DMP1-KI) animals, respectively (Fig. 1a). OB specific activation of IKK2 did not cause any overt phenotype in mice. However, micro-CT scanning data revealed changes in bone parameters in SP7-KI (Fig. 1b–f) and Col1-KI mice (Fig. 1g–k) consistent with osteopenia. DMP1-KI mice also showed moderate decrease in bone mass (BV/TV) (Fig. 1l, m) but no significant changes were observed in other bone trabecular parameters (Fig. 1n–p). These results indicate that the activation of IKK2 during early stages of OB differentiation (SP7-KI and Col1-KI) results in significant decrease in bone mass, while activation in late mature OB and/or early OCy (DMP1-KI) stage results in a moderate decrease in bone mass. In addition to decreased bone mass, histological analyses also revealed a significant reduction in Von-Kossa staining indicative of decreased bone mass and a significant increase in OC number (N.Oc/B.Pm) in SP7-KI mice when compared to WT control animal (Fig. S1A–C). This increase in OC number further suggests that inflammation (through IKK2ca expression in OB cells) can induce OC differentiation and bone loss.

### Deletion of IKK2 at early but not late stages of OB differentiation increases bone mass

**Fig. 2:**
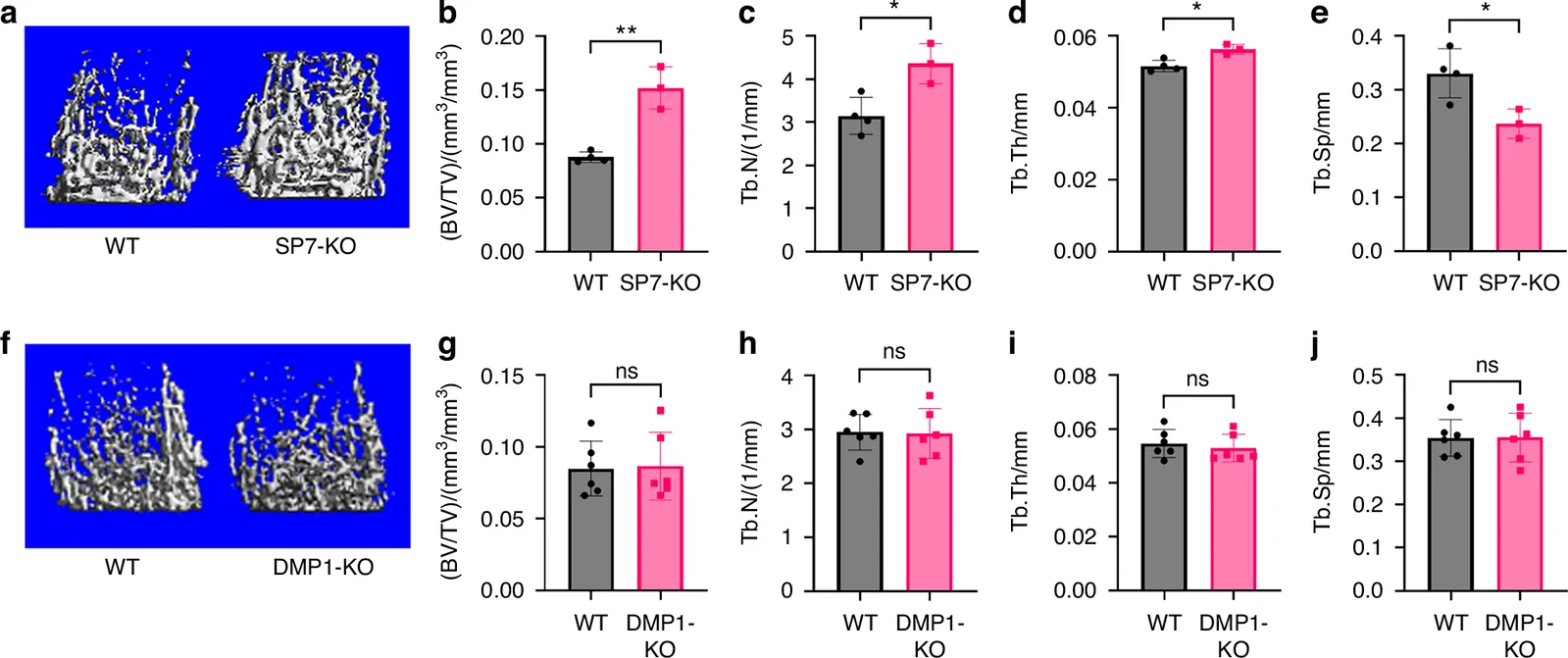
Deletion of IKK2 at early, but not late, stages of OB differentiation increases bone mass. *Sp7*-cre, and *Dmp1*-cre mice were bred with IKK2 f/f mice (*Sp7-Cre:IKK2*^*−/−*^ or SP7-KO) and *Dmp1-Cre:IKK2*^*−/−*^ or DMP1-KO) animals. 6–8 weeks old male and female animals were sacrificed, and long bones (femur) were used for microCT analysis. **a**–**e** MicroCT analysis showing increased bone mass in *Sp7-Cre:IKK2*^*−/−*^ (SP7-KO) compared to littermate WT controls. **f**–**j** There was no significant difference between *Dmp1-Cre:IKK2*^*−/−*^ (DMP1-KO) and control animals. BV/TV (Bone volume/Total volume), Tb.N (Trabecular number), Tb.Th (Trabecular thickness), Tb.Sp (Trabecular Separation). *n* = 3–6 per group. Data show mean ± S.D. *P < 0.05, **P < 0.01 (Student *t* test)

To validate the bone catabolic effect of activated IKK2, we generated loss of function mice by conditionally deleting IKK2 in the OB lineage. To this end, we bred *Sp7-Cre* and *Dmp1-cre* mice with *IKK2−*floxed mice to generate *Sp7-Cre:IKK2*^*−/−*^ knockout (SP7-KO) and *Dmp1-Cre:IKK2*^*−/−*^ knockout (DMP1-KO) animals. SP7-KO mice displayed increased bone mass (BV/TV), trabecular thickness (Tb.Th) and trabecular number (Tb.N) and decreased trabecular separation (Tb.Sp) (Fig. 2a–e) but no differences were observed in DMP1-KO mice compared to their respective littermate WT control mice (Fig. 2f–j). Therefore, inactivation of canonical NF-ĸB signaling through IKK2 deletion at early stage of OB differentiation (SP7-KO) results in an increase in bone mass but does not affect the bone mass at late stage (DMP1-KO). This data suggests that NF-ĸB or NF-ĸB-induced downstream signaling play an important role in differentiation and function of OB to achieve bone mass homeostasis at physiological conditions.

### Inflammation driven by IKK2ca expression induces abnormal differentiation of OB to atypical inflammatory OCy-like cells

**Fig. 3:**
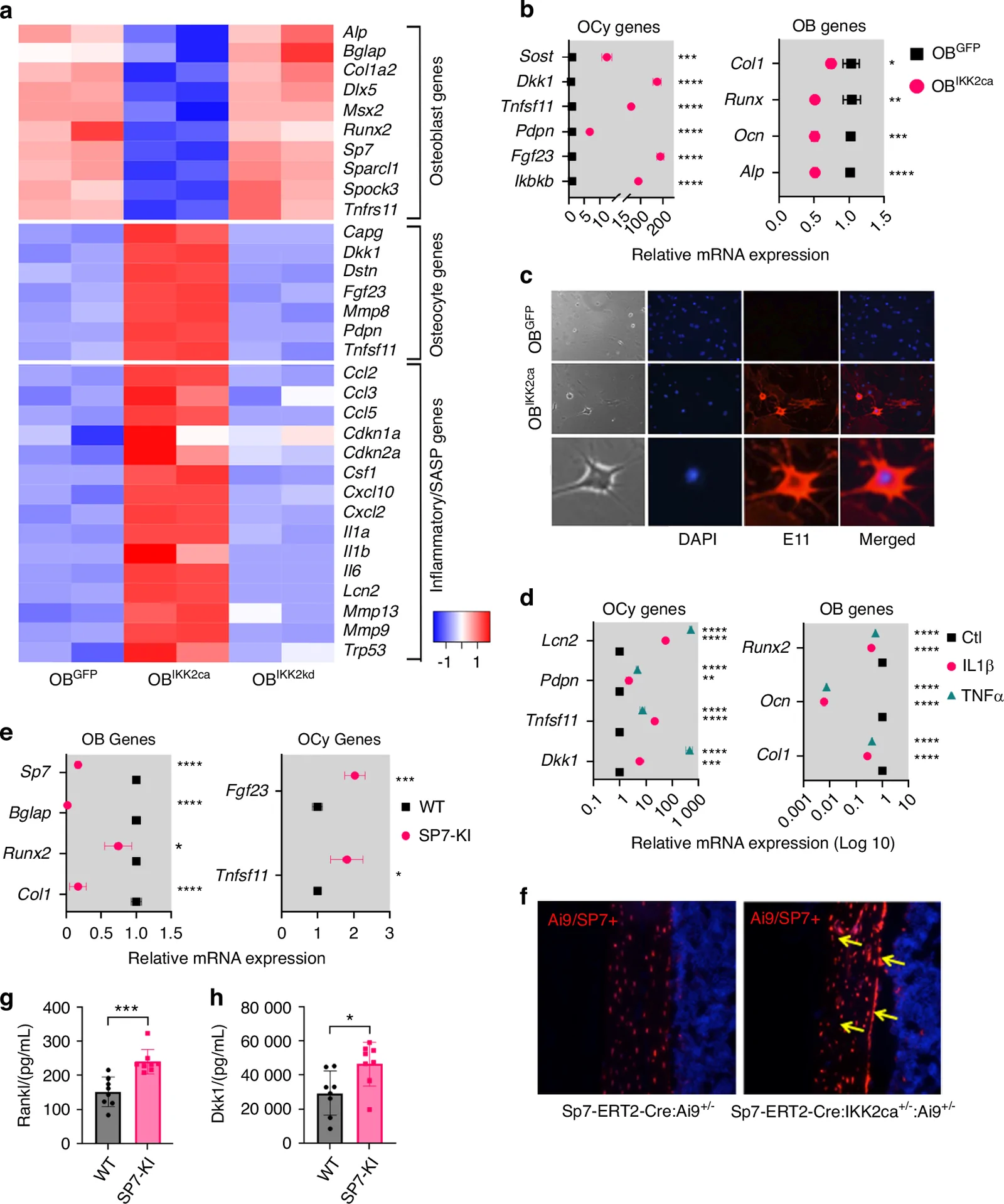
Inflammation driven by IKK2ca expression induces abnormal differentiation of OB to aiOCy-L cells. cOB expressing GFP, IKK2ca and IKK2kd (kinase dead IKK2) were cultured in osteogenic media (OGM: α-MEM supplemented with 10% FBS, 10 mmol/L β-glycerophosphate, 50 µg/mL of ascorbic acid, and 1% penicillin/streptomycin) for 6 days. **a** RNAseq analysis (CPM, scaled by row) shows decreased expression of OB genes (top panel), increased expression of OCy (middle panel) and inflammation/SASPs genes (bottom). **b** qPCR results for selected genes. (**b**: Left panel) shows increased expression of OCy genes. (**b**: Right panel) shows decreased OB’s gene expression. **c** Immunostaining showing osteocytic/dendritic morphology with significantly increased E11 expression in cOB^IKK2ca^ compared to cOB^GFP^ (Lower row is magnification of second row) (**d**) cOBs were treated with IL1β (0.5 ng/mL) and TNFα (0.1 ng/mL) for 6 days. qPCR analysis showed decreased OB and increased OCy gene expression. **e** Bone marrow cells isolated from WT and *Sp7-Cre:IKK2ca*^*+/+*^ (SP7-KI) were differentiated to OB in the OGM containing 10^−7^mol/L dexamethasone for 15 days. qPCR analysis showed decreased OB and increased OCy gene expression. **f** At 6 weeks of age, Cre was activated with Tamoxifen (Tamoxifen diet ad libitum for 2 weeks, Source# Inotive TD.130860) in *Sp7-ERT2-Cre: IKK2ca*^*+/−*^*:Ai9*^*+/−*^ and *Sp7-ERT2-Cre:Ai9*^*+/−*^*)* mice. Immunofluorescence histological images show a higher number of Ai9+ OB/OCy cells in the *Sp7-ERT2-Cre: IKK2ca*^*+/−*^*:Ai9*^*+/−*^ mice compared to control *Sp7-ERT2-Cre:Ai9*^*+/−*^. **g**–**h** Serum ELISA. Data show mean ± S.D. *P < 0.05, **P < 0.01 and ***P < 0.001, ****P < 0.000 1 (Student *t* test). Data is representative of three independent experiments (except RNAseq)

To investigate the molecular mechanism underlying IKK2ca-induced osteopenia, we used primary calvaria OB cells (cOBs) infected with retroviral vectors expressing GFP (denoted cOB^GFP^), IKK2ca (cOB^IKK2ca^) or IKK2kd (kinase dead IKK2; cOB^IKK2kd^), and cultured in osteogenic media for 6 days (Supplementary data 1). RNAseq and qPCR data analyses show significant decrease in OB differentiation-associated genes including *Alp*, *Col1*, *Ocn*, *Tnfsf11b or Opg* and *Runx2/cbfa1* (Fig. 3a top panel and Fig. 3b). Surprisingly, these cells expressed bona fide OCy markers including *Pdpn (E11), Fgf23, Sost, Dkk1 and Tnfsf11a (Rankl*) (Fig. 3a middle panel and Fig. 3b). In addition to OCy markers, these cells displayed increased expression of inflammatory and senescence markers including *Il1β, Tnfα, Il6*, *Cdkn2 (p16), p21, p53*, etc. (Fig. 3a, bottom panel). Supporting the gene expression data, immuno-fluorescence imaging further confirmed high cellular expression of the OCy surface marker E11/podoplanin and an osteocytic/dendritic morphology in cOB^IKK2ca^ (Fig. 3c). To support genetic activation data, we also treated wild type cOB (WT-cOB) with IL1β and TNFα. Similar to cOB^IKK2ca^, we observed decreased expression of OB genes and increased expression of OCy markers (Fig. 3d) in cOB treated with IL1β and TNFα. Based on these observations, we termed these cells as atypical inflammatory osteocyte-like cells (aiOCy-L cells). We also confirmed the inflammation-induced abnormal OB to aiOCy-L phenotype transition by overexpressing p65 and p65-SD (phospho mimetic form of p65) in cOB (Supplementary Data 1). Similar to cOB^IKK2ca^, cOB^p65^ and cOB^p65SD^ cells showed decreased expression of OB genes (Fig. S2A–C), increased expression of OCy genes (Fig. S2D–G) and increased Pdpn/E11 immunofluorescence staining (Fig. S2H). Validating specificity of the effect, cOB^p65SA^ (inactive p65) expression cells did not show any Pdpn Immunostaining (Fig. S3H; bottom panel). Thus, these observations led us to hypothesize that inflammatory signals induce abnormal differentiation of OB to aiOCy-L cells, evident by elevated expression levels of osteocytes, inflammatory, and senescence markers.

To validate our hypothesis in relevant animal model, we used multiple approaches. First, we differentiated bone marrow derived OB from 6 weeks old, WT and SP7-KI mice for 15 days in osteogenic conditions. Consistent with IKK2ca expression and IL1β and TNFα treatment models, bone marrow derived OB from SP7-KI mouse showed decreased expression of OB genes and increased expression of OCy genes compared to WT mouse (Fig. 3e). Second, we generated a *Sp7-ERT2-Cre: IKK2ca*^*+/−*^*:Ai9*^*+/−*^ (KI-Ai9) reporter and control *Ai9*^*(+/-)*^ mice (WT-Ai9). Histological section from these mice showed increased Ai9^+^ cells on bone surface along with disorganized osteocyte network in the cortical bone of KI-Ai9 mice, when compared to WT-Ai9 mice (Fig. 3f). Based on our in vitro observations, we propose that inflammation induces abnormal differentiation of OB to aiOCy-L cells. These cells fail to undergo normal differentiation and cellular function. Consequently, these pathological aiOCy-L cells accumulate near the bone surface failing to embed. Failure to embed in bone is likely a consequence of losing their anabolic phenotype and acquirement of an inflammatory catabolic phenotype that impedes embedding in bone. This phenomenon warrants further investigation in future studies. Finally, serum analysis showed an increased level of Rankl and Dkk1 in SP7-KI mice compared to the WT controls (Fig. 3g, h). Together, the in vitro and in vivo data showed an abnormal differentiation of OB to aiOCy-L cells under inflammation.

### IKK2ca-induced inflammation increases glycolytic metabolism of OBs

**Fig. 4:**
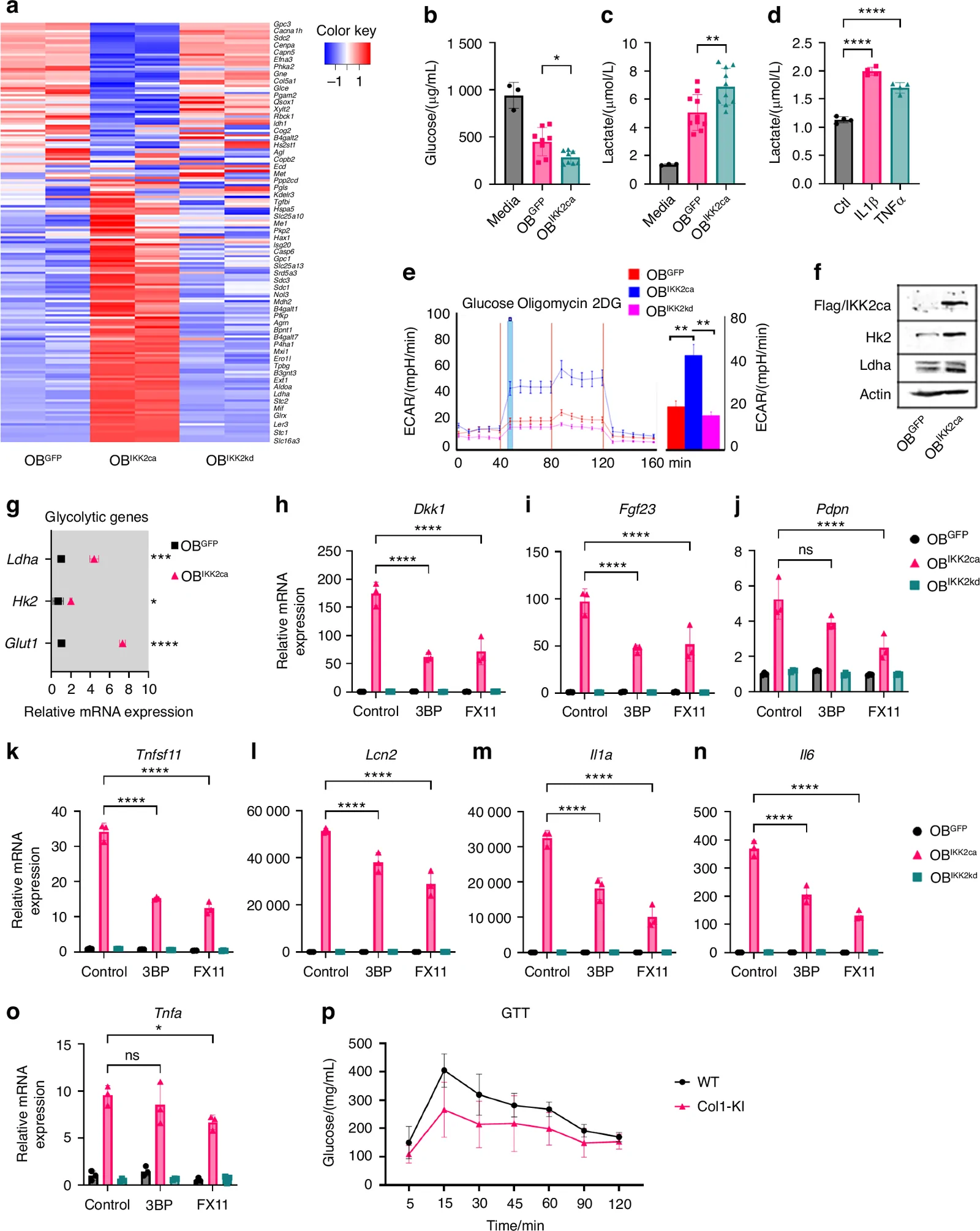
IKK2ca-induced inflammation increases glycolytic metabolism of OBs. cOB expressing GFP, IKK2ca and IKK2kd (kinase dead IKK2) were cultured in osteogenic media (OGM: α-MEM supplemented with 10% FBS, 10 mmol/L β-glycerophosphate, 50 µg/mL of ascorbic acid, and 1% penicillin/streptomycin) for 6 days. Media was changed on 3^rd^ day (**a**) RNAseq analysis showed altered expression of glycolysis genes (Heatmap data was plotted using Limma-Voom transformed counts which are synonymous with log2 counts per million with a prior count of 0.5. These values preserve the mean variance relationship) in cOB^IKK2ca^ cell when compared to cOB^GFP^ and cOB^IKK2kd^. **b** lower glucose level indicating increase glucose consumption and **c** increased lactate production in and cOB^IKK2ca^ cells. **d** cOBs were treated with IL1β (0.5 ng/mL) and TNFα (0.1 ng/mL) for 6 days. Supernatant media showed increased lactate production in cOB treated with IL1β and TNFα. **e** Seahorse metabolic analysis also confirmed increased extracellular acidification rate (ECAR) in cOB^IKK2ca^ compared to control cOB^GFP^ and cOB^IKK2kd^ cells. The ECAR was normalized to total number of cells. **f** Western blot and **g** qPCR showing increased expression of glycolytic genes in cOB^IKK2ca^ cells. cOB^GFP^, cOB^IKK2ca^ and cOB^IKK2kd^ cells were cultured in OGM and treated with or without 3BP (Hk2 inhbitor:100 µmol/L) and FX11 (Ldha inhibitor: 10 µmol/L) for 6 days. qPCR analysis showed that inhibiting Hk2 and Ldha decreased the expression of OCy gene (**h**) *Dkk1*, (**i**) *Fgf23*, (**j**) *Pdpn or E11*, (**k**) *Tnfsf11* or *Rankl* and inflammatory/SASPs (**l**) *Lcn2*, (**m**) *Il1α*, (**n**) *Il6*, and (**o**) *Tnfα*, in cOB^IKK2ca^ cells. **p** GTT analysis showed lower blood glucose or increased glucose consumption in Col1-KI mice compared to littermate WT animals (*n* = 3 per group). Data show mean ± S.D. *P < 0.05, **P < 0.01 and ***P < 0.001, ****P < 0.000 1 (Student *t* test for 2 groups, one-way ANOVA for 3 groups, two way-ANOVA for multiple groups with more than one variable). Data is representative of three independent experiments (except RNAseq)

Along with OCy-like morphology, gene expression, and inflammatory phenotype in OB^IKK2ca^ (aiOCy-L cells), we observed increased acidification of the culture media of OB^IKK2ca^ cells (Fig. S3A), suggesting increased glycolysis. To better understand the effect of IKK2ca, we carried out RNAseq from cOB^GFP^, OB^IKK2ca^, and cOB^IKK2kd^ cells. Transcriptomic data showed altered expression of glycolytic genes (*Glut1, Hk2, Ldha, etc)* in OB^IKK2ca^ cells compared to cOB^GFP^ and cOB^IKK2kd^ (Fig. 4a). Based on these observations and the role of NF-ĸB in regulation of energy metabolism,^29–31^ we examined the metabolic changes in these cells. OB^IKK2ca^ or aiOCy-L cells showed increased glucose consumption (Fig. 4b) and lactic acid production (Fig. 4c) when compared to the cOB^GFP^ after 6 days of culture in osteogenic conditions. Similarly, IL1β and TNFα treatment increased lactate production in cOB (Fig. 4d). These results indicate that IKK2ca expression, which characterizes inflammation, results in increased glucose metabolism in cOB. To confirm the increase in glycolysis, we measured the extracellular acidification rate (ECAR) using Seahorse metabolic analyzer. The cOB^IKK2ca^ cells showed an increase in ECAR when compared with cOB^GFP^ and cOB^IKK2kd^ cells (Fig. 4E). Western blot (Fig. 4F) and qPCR (Fig. 4G) analyses confirmed increased expression of Hk2, Glut1 and Ldha in cOB^IKK2ca^ compared to control cells. Similarly, exogenous expressions of p65 and p65-SD (phospho mimetic form of p65) increased glycolytic genes (Figure S3B-D). Further, exposure to Hk2 inhibitor (3-BP) and Ldha inhibitor (FX11) significantly inhibited the expression of OCy genes including *Dkk1*, *Fgf23* (Fig. 4h, i), as well as *Rankl* (Fig. 4k), and the inflammatory senescence-associated secretory phenotype markers (SASPs) *Lcn2, Il1α* and *Il6* (Fig. 4l–n) in cOB^IKK2ca^ cells. Moreover, FX11 but not 3-BP, significantly inhibited the expression of *Pdpn/*E11 (Fig. 4j) and *Tnfα* (Fig. 4o) in cOB^IKK2ca^ cells. In addition, treatment with Hk2 inhibitor (3-BP) and Ldha inhibitor (FX11) rescued the OCy dendritic/morphology in cOB^IKK2ca^ cell (Fig. S3E). There was no significant decrease in expression of *Alp*, *Col1, Opg* and *Runx2* in cOB^IKK2ca^ treated with glycolytic inhibitor when compared with cOB^IKK2ca^ (Fig. S3F–I). Finally, using glucose tolerance test (GTT), we provide evidence that serum glucose levels in IKK2ca expressing mice are markedly reduced (20%-25%) compared with WT littermates, indicating increase in glucose consumption (Fig. 4p). Together these data show that inflammation regulates glycolytic metabolism in OB, which results in abnormal differentiation of OB to aiOCy-L cells.

### IKK2ca-induced abnormal differentiation of OB to atypical inflammatory OCy-like cells is mediated through mTOR activation

**Fig. 5:**
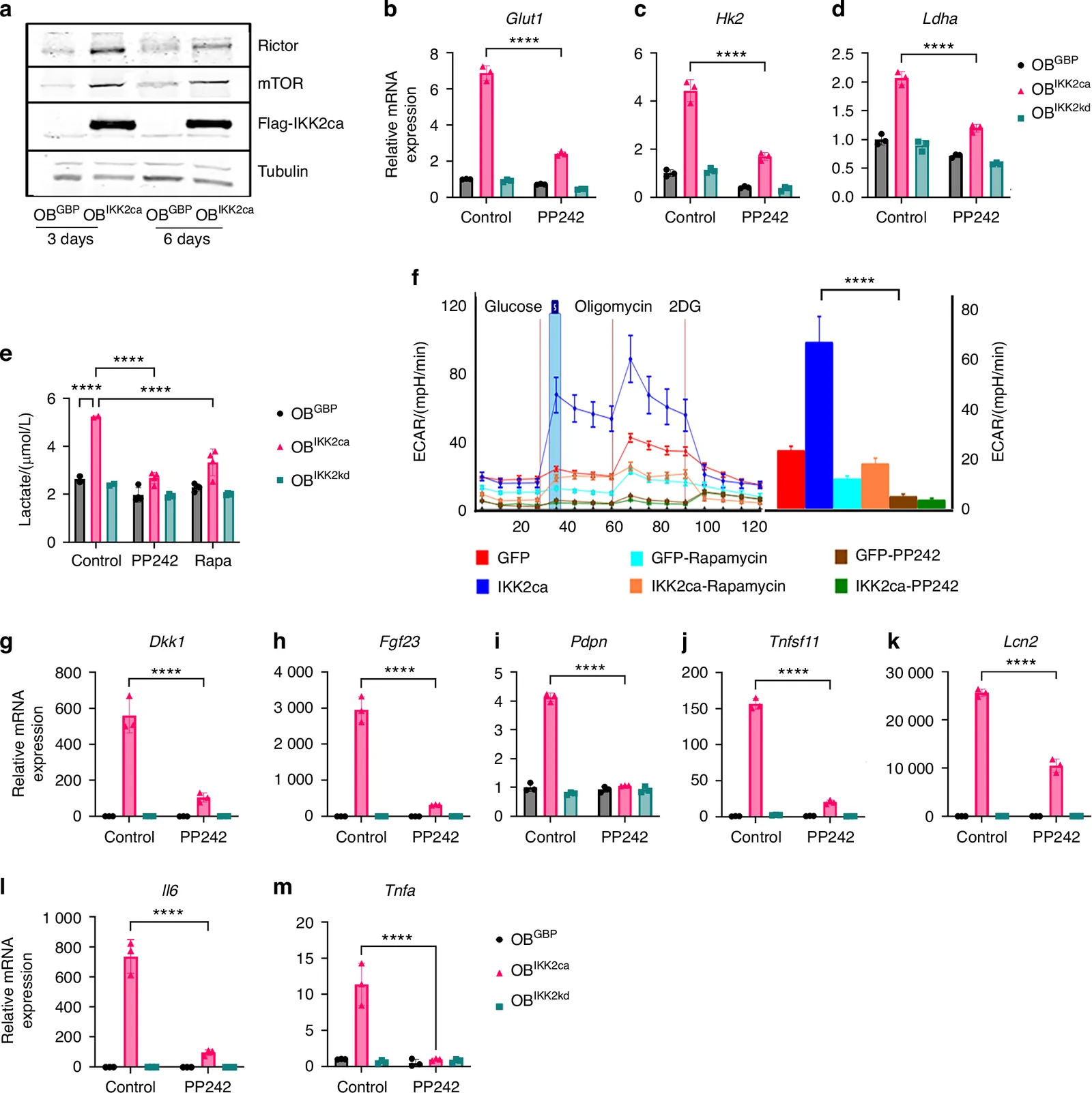
IKK2ca-induced abnormal differentiation of OB to aiOCy-L cells is mediated through mTOR activation. cOB expressing GFP, IKK2ca and/or IKK2kd (kinase dead IKK2) were cultured in osteogenic media (OGM: α-MEM supplemented with 10% FBS, 10 mmol/L β-glycerophosphate, 50 µg/mL of ascorbic acid, and 1% penicillin/streptomycin) for 3 and/or 6 days with or without mTOR1/2 inhibitor PP242 (and/or rapamycin) at 1 µmol/L concentration. WB analysis shows increased mTOR activation in (**a**) cOB^IKK2ca^ cells. qPCR analysis showed that mTOR signaling regulates glycolysis, evident by significant decreased **b** *Glut1*, **c** *Hk2* and **d** *Ldha* expression in cOB^IKK2ca^ cells treated with PP242 (Torin). **e** Decreased lactate secretion and **f** decreased ECAR (normalized to total number of cells) in cOB^IKK2ca^ cells treated with mTOR1/2 inhibitors further showed mTOR mediated regulation of glycolysis in cOB^IKK2ca^ cells. qPCR showed decrease expression of OCy genes **g** *Dkk1*, **h** *Fgf23*, **i** *Pdpn* and **j** *Tnfsf11* or *Rankl* and inflammatory/SASPs genes **k** *Lcn2*, **l** *Il6* and **m** *Tnfα*, in cOB^IKK2ca^ cells treated with mTOR inhibitors. Data show mean ± S.D. *P < 0.05, **P < 0.01 and ***P < 0.001, ****P < 0.000 1 (Student *t* test for 2 groups, one-way ANOVA for 3 groups, two way-ANOVA for multiple groups with more than one variable). Data is representative of three independent experiments

Since we observed an abnormal differentiation of OB to aiOCy-L cells along with increased glycolysis, we further explored potential mechanistic targets of this phenomenon. mTOR signaling, specifically in OB and bone development, has been shown to regulate various cellular functions including proliferation, differentiation, cytoskeletal rearrangement and most importantly cellular metabolism.^32–35^ Additionally, several reports suggest that IKK2/NF-ĸB mediates regulation of mTOR signaling and cellular function in various other cells.^36–38^ Western blot data show increased mTOR and Rictor expression in OB^IKK2ca^ cells (Fig. 5a) indicating that IKK2 regulates mTOR signaling. TNFα treatment also increased mTORC2 downstream p-Akt473/total-Akt expression (Fig. S4A). We further cultured cOB^GFP^, OB^IKK2ca^, and cOB^IKK2kd^ cells under osteogenic conditions in the presence or absence of mTOR specific inhibitor PP242 (inhibits both mTORC1 and mTORC2) or Rapamycin. The qPCR results show that mTOR inhibition significantly decreased the expression of glycolysis genes *Hk2*, *Glut1* and *Ldha* in OB^IKK2ca^ cells (Fig. 5b–d). Treatment of OB^IKK2ca^ cells with mTOR inhibitors also decreased lactate levels (Fig. 5e) and ECAR (Fig. 5f), suggesting that IKK2ca induced mTOR activation regulates glycolysis in OB^IKK2ca^ cells. Finally, we investigated if mTOR inhibition can rescue differentiation of OB to aiOCy-L cells. The data show a decrease in the osteocyte/dendritic morphology of OB^IKK2ca^ (aiOCy-L) cells that were treated with mTOR inhibitors (Fig. S4B). Consistently, qPCR results show that mTOR inhibition significantly decreased the expression of OCy genes like *Dkk1*, *Fgf23*, *Rankl* and *Pdpn* (Fig. 5g–j) and inflammatory/SASP genes like *Lcn2, Il6 and Tnfα* (Fig. 5k–m) in OB^IKK2ca^ cells with no decrease in expression of OB specific genes *Col1* and *Runx2* (Fig. S4C, D). These results indicate that inflammation mediated by IKK2 activation in cOB activates mTOR, which in turn regulates glycolysis and leads to abnormal differentiation of OB to aiOCy-L cells.

## Discussion

Inflammation associated with conditions such as rheumatoid arthritis, osteoarthritis or metabolic diseases promotes the release of numerous factors that alter OB function and impair bone formation, while increasing bone resorption. Understanding the molecular pathways through which inflammation disrupts OBs, is therefore essential for developing strategies to preserve OB function and promote bone health.

Several inflammatory mediators, including TNFα, IL1β, IL6, and IL17 inhibit bone formation, thereby complicating therapeutic interventions. Although these mediators have distinct signaling mechanisms, they commonly require activation of the NF-κB pathway to exert their effect. Leveraging this observation, we utilized a constitutive active genetic IKK2ca expression model to activate NF-κB, thereby mimicking inflammation specifically within OB cells.

MicroCT analysis of mice with IKK2ca driven by early OB-specific Cre recombinases (*Sp7* and *Col1*-cre) revealed significant bone abnormalities. In contrast, when IKK2ca expression was induced in late OBs or early OCy using *Dmp1*-cre, there was only a moderate decrease in bone mass. This indicates that bone formation by OBs is more susceptible to inflammation induced disruption during early differentiation stages compared to late maturation stages. Additionally, deleting IKK2 at an early stage using *Sp7*-cre resulted in increased bone mass, whereas deleting IKK2 at a late stage of OB differentiation using *Dmp1*-cre did not show a significant increase in bone mass. These findings suggest that NF-κB, or the signaling pathways it induces, play a crucial role in OB differentiation and function, which is essential for maintaining basal bone mass under physiological conditions.

At the molecular level, cOBs expressing GFP, IKK2ca, or IKK2kd (kinase-dead IKK2), or treated with IL1β and TNFα displayed a significant reduction in the expression of genes associated with OB differentiation. Most notably, these cells adopted osteocytic/dendritic morphology and expressed OCy genes. These OCy-like cells also exhibited increased expression of inflammatory and senescence markers. While it is generally accepted that under physiological conditions most OBs undergo apoptosis, some become embedded in the bone matrix and further differentiate into OCy, a phenomenon defined as osteocytogenesis. Our results indicate that inflammatory responses abnormally alter the OB differentiation program and fate decisions, leading to their differentiation into aiOCy-L cells. These aiOCy-L cells are poorly embedded in the bone matrix and predominantly clustered at the bone lining interface. Although we are not claiming that this inflammatory OB phenotype is OCy, nevertheless these aiOCy-L cells probably represent inflammation induced abnormal inflammatory OB-derived phenotype, where OB fails to undergo normal development, differentiation and cellular function. These findings present a fundamental shift in the understanding of OB differentiation and function under inflammatory conditions and offer opportunities for future investigations.

The aiOCy-L cells express elevated levels of Dkk1 and Sost, that directly impede OB differentiation. Concurrently, the higher expression of Rankl along with SASPs and inflammatory cytokines enhances osteoclast differentiation. Collectively, these processes create a vicious cycle where aiOCy-L cells simultaneously inhibit OB activity and promote osteoclast activity, resulting in overall bone loss and osteopenia. This is consistent with and supports numerous clinical and animal studies showing increased serum RANKL, FGF23, DKK1 and SOST expression in inflammatory diseases such as inflammatory bowel diseases (IBD), arthritis, ageing and cancer. These observations buttress the significance of our findings that inflammatory signals induce abnormal differentiation of OB to aiOCy-L cells resulting in net bone loss.^39–44^ In our ongoing and future research, using various inflammatory bone loss models, we are investigating the detailed molecular signaling mechanism through which NF-ĸB activation induces metabolic changes that subsequently regulate the abnormal differentiation of OB to aiOCy-L cells. For instance, we are examining the role of aiOCy-L cells in ageing associated bone loss, where multiple studies have shown increased expression of inflammatory and senescence associated genes.^45,46^ Similarly, aiOCy-L cells may be crucial to understand the absence of adequate new bone formation and repair in the arthritic joints.^47^ This is because the inflammatory milieu in joints can promote OB to aiOCy-L differentiation, which subsequently inhibit OB function leading to a lack of new bone formation. Thus, characterization and detailed signaling analysis of aiOCy-L cells can pave the way to target bone loss in various inflammatory disorders.

Previous studies have identified mTOR signaling and glycolytic metabolism as important regulators of physiological OB differentiation and function.^32–35,48,49^ For example, deletion of Raptor (component of mTORC1) in mouse leads to osteopenia. Similarly, Rictor (mTORC2 component) deficient mouse shows impaired bone formation. Other studies have shown OB differentiation relies heavily on glycolytic metabolism.^34^ However, in the context of inflammation, we provide evidence that these pathways are aberrantly upregulated in OBs resulting in the aiOCy-L cell phenotype. Accordingly, inflammation seems to highjack these physiologically essential signaling pathways in OB, diverting them towards an abnormal OB derived inflammatory phenotype. Using glycolytic and mTOR inhibitors we further showed that inhibiting these elevated pathways to physiological level can inhibit this abnormal differentiation of OB, without negatively regulating any OB anabolic genes. The reversal of this abnormal phenotype at the molecular and cellular levels following inhibition of mTOR and glycolysis suggests a temporal hierarchy wherein inflammation induced-mTOR and glycolysis mediates abnormal gene expression during this process.

Our data does not specify the relative contributions of mTORC1 and mTORC2. We observed increased expression of Rictor, a component of mTORC2, and found that PP242, a pan-mTOR inhibitor, has a stronger effect than rapamycin, which primarily inhibits mTORC1. This dichotomy can be explained by the fact that, with prolonged exposure such as 3–6 days in our experiments, rapamycin indirectly inhibits mTORC2 by preventing the assembly of new mTORC2 complexes. We believe that rapamycin first inhibits mTORC1 and later mTORC2, whereas PP242 inhibits both mTOR complexes simultaneously, resulting in a stronger inhibitory effect. We performed these studies using pharmacological inhibitors in lieu of genetic intervention, to avoid complete inhibition of mTOR or glycolysis which can negatively regulate the OB differentiation and function irrespective of inflammation.

In conclusion, our study explores how inflammation alters OB function, with a focus on NF-κB signaling. Using in vivo and in vitro models, we demonstrated that inflammatory mediators cause OBs to differentiate into aiOCy-L cells, which exhibit increased expressions of OCy genes and SASPs, negatively impacting bone formation and promoting OC differentiation. Our findings align with clinical observations of elevated OCy specific proteins in various inflammatory conditions and provide novel insights into the molecular pathways driving inflammation-induced bone loss. The identification of aiOCy-L cells challenges traditional understanding of OB differentiation concepts and suggests that targeting mTOR signaling and glycolytic metabolism could inhibit the abnormal differentiation of OBs, preserving their function and promoting bone health. This could lead to new therapies for managing and preventing bone loss diseases associated with inflammation.

## Material and methods

### Animals

All experiments were performed using littermate control mice. Mice were housed at the Washington University School of Medicine barrier facility with 12 h light/dark cycles. All experimental protocols were carried out in accordance with the ethical guidelines approved by the Washington University School of Medicine Institutional Animal Care and Use Committee (IACUC). In order to conditionally activate IKK2/NF-ĸB in OB, we crossed *Sp7*-cre,^50^ *Col1cre*-cre^51^ and *Dmp1*-cre^52,53^ mice with *IKK2ca* floxed^54^ mice to generate *Sp7-Cre:IKK2ca*^*+/+*^ (SP7-KI), *Col1-Cre:IKK2ca*^*+/+*^ (Col1-KI) and *Dmp1-Cre:IKK2ca*^*+/+*^ (DMP1-KI) animals respectively. Similarly, to conditionally inactivate NF-ĸB signaling in OB we crossed *Sp7*-cre, and *Dmp1*-cre mice with IKK2-f/f mice^55^ (*Sp7-Cre:IKK2*^*−/−*^ or SP7-KO and *Dmp1-Cre:IKK2*^*−/−*^ or DMP1-KO) animals. To generate *Sp7-ERT2-Cre: IKK2ca*^*+/−*^*:Ai9*^*+/−*^reporter mice, we first crossed *Sp7cre-ERT2-cre*^56^ mice with *IKK2ca* floxed mice. *Sp7cre-ERT2-cre:IKK2ca*^*+/)*^ mice were then bred with *Ai9*^*+/)*^ (Jax Strain# 007909) animals. Since both Ai9 and IKK2ca genes are engineered at ROSA26 loci (Chr6), we generated animals with only one allelic copy of Ai9 and IKK2ca (*Sp7-ERT2-Cre: IKK2ca*^*+/−*^*:Ai9*^*+/−*^*)* as two copies of 2 genes at same loci is not feasible. At 6 weeks of age, Cre was activated with Tamoxifen (Tamoxifen diet ad libitum for 2 weeks, Source# Inotive TD.130860). Wild type (WT) mice were generated by breeding C57BL/6 animals (for cOB culture).

### MicroCT analysis

For microCT analysis, animals (male and female) were sacrificed between 7 and 8 weeks of age. Intact long bones (femur/tibia) were isolated, fixed overnight in 10% neutral buffered formalin followed by washing with phosphate buffer saline (PBS) two times and transferred to 70% ethanol (v/v). Bones were then scanned using Scanco Medical micro-CT systems (Scanco, Wayne, PA, USA) at the core facility at the Musculoskeletal Center at Washington University in St. Louis (St. Louis, MO). Bones were scanned at a resolution of 20 µm, slice increment 20 µm, voltage 55 kV, current 145 µA and exposure time of 200 ms. After scanning, contours were drawn from the growth plate toward trabecular regions of femur. Approximately 150 slices were analyzed.

### Cell culture

For primary OB culture, calvaria were collected from neonatal mice pups (1–2 days old from WT animals).^28^ Specifically, calvaria were surgically removed from the skull, sutures were removed, and adherent tissue was cleaned by gentle scrapping using a sterilized scalpel, followed by cutting the calvaria to very small fragments. These small chopped calvaria tissue was digested (5 time; 15 min/digestion) with 0.1% dispase and 0.1% collagenase P to release cells. After discarding the cells from the first digestion (heterogeneous population), cells from the next four digestions were pooled and filtered using 70 μm strainers. The cells were centrifuged at 1 500 RPM for 5 min followed by culturing in OB proliferation media (ascorbic acid free-modified α-MEM) containing 10% FBS. For OB differentiation, the cOBs were cultured in osteogenic media (α-MEM supplemented with 10% FBS, 10 mmol/L β-glycerophosphate, 50 µg/mL of ascorbic acid, and 1% penicillin/streptomycin).

For OB culture from bone marrow cells, total bone marrow was flushed out and seeded at a density of 2 × 10^6^ cells/well of 12-well plates for 15 days in osteogenic media containing 100 nmol/L dexamethasone.

### Retroviral infection in primary cOB

Retroviral particles were generated by transfecting pMX-GFP, pMX-IKK2ca, pMX-IKK2kd, pMX-p65 (human), pMX-p65SD (human) and pMX-p65SA (human) into PLAT-E cell (Cellbio Labs) using Xtremegene-9 transfection reagent.^28^ The insert sequences are described in supplemental data 1. cOBs were infected with retroviral particles in OB proliferation media for 12–24 h, followed by culturing according to the experiment conditions.

### RNA sequencing and analysis

Primary Calvaria OBs were infected with retroviral vector expressing GFP (cOB^GFP^), IKK2ca (cOB^IKK2ca^) and IKK2kd (cOB^IKK2kd^) followed by culture in osteogenic media for 6 days. Samples were processed at Genome Technology Access Centre (GTAC) core at Washington University. Specifically, total RNA integrity was determined using Agilent. Library preparation was performed with 5 μg of total RNA with a Bioanalyzer RIN score greater than 8.0. Ribosomal RNA was removed by poly-A selection using Oligo-dT beads (mRNA Direct kit, Life Technologies). mRNA was then fragmented in reverse transcriptase buffer and heated to 94 degrees for 8 min. mRNA was reverse transcribed to yield cDNA using SuperScript III RT enzyme (Life Technologies, per manufacturer’s instructions) and random hexamers. A second strand reaction was performed to yield ds-cDNA. cDNA was blunt ended, had an A base added to the 3’ ends, and then had Illumina sequencing adapters ligated to the ends. Ligated fragments were then amplified for 12–15 cycles using primers incorporating unique dual index tags. Fragments were sequenced on an Illumina NovaSeq 2500 using single end reads extending 50 bases. RNAseq analysis was performed as described earlier.^31^

### Realtime PCR

RNA was isolated from different cells post treatments using PureLink RNA mini kit (Cat# 12183025, Ambion, Grand Island, NY, USA) and cDNA was synthesized using High-Capacity cDNA Reverse Transcription kit (Cat# 4368814, Applied Biosystems). Realtime PCRs were carried out on BioRad CFX96 real time system using iTaq universal SYBR green super-mix (Cat#1725120, BioRad, Hercules, CA, USA). mRNA expression was normalized using *Actin* as a housekeeping gene. The primers are listed in Table S1.

### Western blotting

Total cell lysates for protein analysis were collected by scraping cells in 1x Cell Lysis Buffer (Cell Signaling Technology, Danvers, MA, USA) containing protease and phosphatase inhibitor cocktail (ThermoFisher Scientific, Halt Protease Phosphatase Inhibitor Cocktail). Protein concentration was determined by rapid BCA assay kit (Pierce) and equal amounts of proteins were used for gel electrophoresis (SDS-PAGE). After wet transfer to nitrocellulose membrane using BioRad Transfer system using 1x transfer buffer. Membranes were blocked with 5% BSA in PBS-Tween (0.1% v/v) (PBST) for 1 h at room temperature, and probed with specific primary antibodies diluted in 5% BSA in PBST overnight at 4 °C. Following Primary antibodies were used: Flag (Cat#F3165, Sigma, St. Louis, MO), Hk2 (Cat#2687, Cells Signaling Technology), Ldha (Cat#3582, Cells Signaling Technology), Glut1 (Cat#12939, Cells Signaling Technology), mTOR (Cat#2983, Cells Signaling Technology, Rictor (Cat#2114, Cells Signaling Technology, Actin (Cat#A2228, Sigma, St. Louis, MO). All primary antibodies were used at a 1:1 000 dilution, except for Actin, which was used at a 1:10 000 dilution. Next day, membranes were washed with PBST (3 times) and probed with secondary antibodies from LI-COR (Odyssey Imager; donkey anti-rabbit/IRDye, 800CW/anti-goat/IRDye 800CW, anti-mouse IRDye 680RD, Donkey anti-rat/IRDye 800CW, 1:10 000 dilution) for 1 h at room temperature. Membranes were then washed in PBST (3 times, 10 min each time) and scanned using LI-COR Odyssey Imager (LI-COR Biosciences, Lincoln, NE, USA) at low resolution using Licor software.

### Immunofluorescence staining

Pdpn or E11 has been described as a marker for osteocytes,^57^ which is expressed during dendritic elongation. cOB^GFP^, cOB^IKK2ca^, cOB^p65^, cOB^p65SD^ and cOB^p65SA^ cells were fixed with 4% paraformaldehyde in PBS for 15 min followed by washing with 1x PBS. The cells were then blocked for 1 h at room temperature using: PBS **+** 10% goat serum (HyClone Laboratories). After blocking, primary antibody, Podoplanin in PBS **+** 3% goat serum (8.1.1; Santa Cruz Biotechnology, Santa Cruz, CA, USA) was added (1:100 dilution, overnight at 4 °C). Next day the cells were washed three times with PBS and incubated with secondary antibody-Alexa Fluor 594 labeled goat anti-hamster IgG (Molecular Probes) at 10 μg/mL for 45 min at room temperature. Cells were then washed with PBS, counter-stained with 4′,6-diamidino-2-phenylindole (DAPI; Vector) and mounted prior to imaging by fluorescence microscopy.

### Measurement of cellular metabolism by Seahorse

cOB^GFP^, cOB^IKK2ca^ and cOB^IKK2kd^ were plated in Seahorse XF96 plates at 5 000 cells per well in OGM and treated with or without mTOR inhibitor rapamycin (1 μmol/L) and PP242 (1 μmol/L) for 6 days. Seahorse assay was performed after 6 days. For glycolysis stress test, cells were starved for 1 h in glucose-free media containing treatments, and measurement of ECAR was performed prior to and after sequential addition of glucose, oligomycin and 2-DG with measurements performed every 5 min. The ECAR measurements were normalized using cell numbers. It is important to note that cells were cultured for 6 days where they form a uniform monolayer in the culture plates with similar number of cells. Data was analyzed using Wave software.

### Glucose and Lactate measurement in media supernatant

cOB^GFP^, cOB^IKK2ca^ and cOB^IKK2kd^ were cultured in OGM and treated with or without mTOR inhibitor rapamycin (1 μmol/L) and PP242 (1 μmol/L) for 6 days. Supernatant media was collected and centrifuged to separate cell debris and floating cells. Supernatant was used immediately or stored at **−**80 °C. Lactic acid assay was performed to measure secreted lactate in the media using Lactate Assay Kit (Cat# 1200011002, Eton Biosciences, l-Lactate Assay Kit I). For glucose consumption measurements were performed with Glucose (HK) Assay Kit (Sigma cat# GAHK20) and read at 340 OD using a plate reader (BioTek, Gen5 software). Unconditioned OGM with 10% FBS was used as a control for subtracting background.

### ELISA

Serum from WT and SP7-KI mice were subjected to ELISA to measure serum Rankl (R&D, Cat # MTR00) and Dkk1 (R&D, Cat # MKK100) as per manufacturer’s instructions.

## Supplementary information

Supplementary Data 1

Supplementary Table 1

Supplementary Figures

## Source data

Source Data

## Data Availability

All data are available in the main text and supplementary materials.
