# Supplementary material for: Inflammation driven by NF-ĸB compromises bone formation by inducing the differentiation of osteoblasts into atypical inflammatory osteocyte-like cells
Source: Bone Res. 2026 Aug 4;14:80. doi: 10.1038/s41413-026-00546-0 (PMC13439236; doi:10.1038/s41413-026-00546-0)
Supplement: Supplementary file 1 [file 41413_2026_546_MOESM1_ESM.docx]

**The Role of the IκB Kinase Complex and Constitutively Active Variants in NF-κB Signaling.**

The IκB kinase (IKK) complex, crucial for NF-κB activation, phosphorylates IκB inhibitors, leading to their degradation. The complex includes IKKα (IKK1), IKKβ (IKK2), and NEMO (IKKγ). When activated by stimuli such as inflammatory cytokines or stress signals, IKK2 phosphorylates IκBα, resulting in its degradation and release of p65/p50 dimers, which then activate target genes in the nucleus.

IKK2ca is created by substituting two serine residues with glutamic acid in the activation loop (S177E and S181E), enabling it to constantly phosphorylate IκBα, leading to its degradation and consequent activation of p65, even without upstream signals. Conversely, IKK2kd, a kinase-dead variant, is formed by substituting lysine with alanine in the ATP binding pocket (K44A), preventing its phosphorylation and thus inhibiting downstream activation of NF-κB signaling. As the final step in NF-κB signaling, p65 is phosphorylated and moves to the nucleus, where it activates gene transcription. Thus, overexpression of IKK2ca or phosphorylated p65 can lead to NF-κB activation without any inflammatory stimuli. p65-SD refers to a specific [phosphomimetic mutant](https://www.google.com/search?q=phosphomimetic+mutant&sca_esv=492d03d456b59a14&rlz=1C1GCEB_en&sxsrf=ANbL-n6iBm07RpcLA5EvDHEDzNt0DByA0w%3A1768503432345&ei=iDhpacjnFIjfkPIPwrDxiQw&ved=2ahUKEwjquPfdnI6SAxXvIUQIHTDINVAQgK4QegQIARAC&uact=5&oq=p65-S536D&gs_lp=Egxnd3Mtd2l6LXNlcnAiCXA2NS1TNTM2RDIFEAAY7wUyBRAAGO8FMggQABiABBiiBDIFEAAY7wVIvA1QAFgAcAB4AZABAJgBjwGgAY8BqgEDMC4xuAEDyAEA-AEBmAIBoAKTAZgDAJIHAzAuMaAH_AGyBwMwLjG4B5MBwgcDMC4xyAcCgAgB&sclient=gws-wiz-serp) of the [NF-κB p65 protein (RelA)](https://www.google.com/search?q=NF-%CE%BAB+p65+protein+%28RelA%29&sca_esv=492d03d456b59a14&rlz=1C1GCEB_en&sxsrf=ANbL-n6iBm07RpcLA5EvDHEDzNt0DByA0w%3A1768503432345&ei=iDhpacjnFIjfkPIPwrDxiQw&ved=2ahUKEwjquPfdnI6SAxXvIUQIHTDINVAQgK4QegQIARAD&uact=5&oq=p65-S536D&gs_lp=Egxnd3Mtd2l6LXNlcnAiCXA2NS1TNTM2RDIFEAAY7wUyBRAAGO8FMggQABiABBiiBDIFEAAY7wVIvA1QAFgAcAB4AZABAJgBjwGgAY8BqgEDMC4xuAEDyAEA-AEBmAIBoAKTAZgDAJIHAzAuMaAH_AGyBwMwLjG4B5MBwgcDMC4xyAcCgAgB&sclient=gws-wiz-serp), where Serine 536 is mutated to Aspartic Acid (D).

**Retro viral Plasmids.**  pMX-GFP, pMX-IKK2ca, pMX-IKK2KD, pMX-p65 and pMX-p65SD were generated by cloning the construct in PMX-Puro retroviral vector (cat# RTV-012 Cell Biolabs, Inc). The sequence with mutation (Red) and Tag (Underline) are shown below. For cloning pMX-IKK2ca and IKK2kd, we used EcoR1 and Not1 restriction sites. For pMX-p65 and pMX-p65SD we used EcoR1 and Xba1 restriction sites. Both pMX-p65 and pMX-p65SD are human sequences.

**pMX-IKK2ca:** ATGGACTACAAAGACGATGACGACAAGAGCTGGTCACCGTCCCTCCCAACCCAGACATGTGGAGCCTGGGAAATGAAAGAACGCCTGGGGACCGGGGGATTTGGAAACGTCATCCGGTGGCACAATCAGGCGACAGGTGAACAGATCGCCATCAAGCAATGCCGACAGGAGCTCAGCCCAAAGAACAGAGACCGCTGGTGCCTCGAAATCCAGATCATGAGAAGGCTGAACCATCCCAATGTGGTGGCTGCCCGGGATGTCCCAGAGGGGATGCAGAACCTGGCACCCAATGATTTGCCACTGCTGGCCATGGAGTACTGCCAAGGAGGAGATCTCCGAAGATACTTGAACCAGTTCGAGAACTGCTGTGGCCTGCGGGAAGGAGCTGTCCTTACCCTGCTGAGTGACATCGCATCGGCTCTTAGATACCTTCACGAAAACAGAATCATCCATCGAGACCTGAAGCCAGAAAACATCGTTCTGCAGCAAGGAGAGAAAAGATTAATACACAAAATTATTGATCTAGGATATGCCAAGGAGCTGGATCAGGGCGAACTGTGCACGGAATTTGTGGGGACTCTGCAATACCTGGCGCCAGAGCTTCTGGAGCAGCAGAAGTACACCGTGACCGTTGACTACTGGAGCTTCGGCACCCTGGCCTTCGAGTGCATCACTGGCTTCCGGCCCTTCCTCCCTAACTGGCAGCCTGTGCAGTGGCACTCCAAAGTCCGGCAGAAGAGCGAAGTGGACATCGTTGTTAGTGAAGACTTGAATGGAGCAGTGAAGTTTTCAAGTTCGCTACCCTTCCCCAATAATCTTAACAGTGTCTTGGCTGAACGGCTGGAGAAGTGGCTGCAGCTGATGCTTATGTGGCACCCTCGGCAAAGGGGCACGGATCCCCAGTATGGCCCCAACGGCTGCTTCAGAGCCCTGGATGACATCTTGAACTTGAAGCTGGTTCATGTCTTGAACATGGTCACAGGCACCGTTCACACATACCCCGTGACGGAGGATGAGAGTCTGCAGAGCTTAAAAACCAGAATCCAGGAAGACACGGGGATCCTGGAGACAGACCAGGAGCTGCTGCAAGAGGCAGGGCTGGTGCTGCTCCCTGACAAGCCTGCTACTCAGTGCATCTCAGACAGCAAGACAAACGAGGGCCTCACGTTGGACATGGATCTTGTTTTTCTCTTTGACAACAGTAAAATCAACTATGAGACTCAGATCACCCCCCGACCCCAACCGGAAAGTGTCAGCTGTATCCTTCAGGAGCCCAAGCGGAACCTCTCCTTCTTCCAGCTGAGGAAAGTGTGGGGCCAAGTCTGGCACAGCATCCAGACGCTGAAGGAAGACTGTAACCGGCTGCAGCAGGGACAGCGAGCAGCCATGATGAGTCTCCTCCGGAATAACAGCTGCCTCTCTAAGATGAAGAACGCCATGGCCTCCACGGCCCAGCAGCTCAAGGCCAAGCTGGACTTCTTCAAAACCAGCATCCAGATCGACCTGGAGAAGTATAAAGAGCAGACCGAGTTTGGGATCACCTCAGATAAATTGCTGCTGGCTTGGCGGGAGATGGAGCAGGCTGTGGAGCAGTGTGGGCGGGAGAATGACGTGAAGCATCTAGTAGAGCGGATGATGGCACTGCAGACTGACATTGTGGACCTGCAGAGGAGCCCGATGGGTCGGAAGCAGGGGGGCACCCTGGATGACCTAGAGGAACAAGCGAGGGAGCTCTACCGAAGACTCAGGGAGAAGCCAAGAGACCAAAGGACAGAAGGTGACAGCCAGGAGATGGTACGGCTGCTGCTTCAGGCAATCCAAAGCTTTGAGAAGAAAGTTCGGGTGATTTATACACAGCTCAGTAAGACCGTGGTTTGTAAGCAGAAGGCACTGGAGTTGCTGCCCAAGGTAGAAGAGGTAGTGAGCCTTATGAACGAGGACGAGAGGACCGTGGTCCGGCTTCAGGAGAAGCGGCAGAAGGAACTCTGGAACCTCCTGAAGATCGCCTGTAGCAAAGTCCGAGGTCCCGTGAGTGGAAGCCCAGACAGCATGAATGTGTCTCGACTCAGTCACCCTGGTCAGCTAATGTCCCAGCCTTCCAGTGCCTGTGACAGCTTACCTGAATCAGACAAGAAAAGTGAAGAACTGGTGGCCGAAGCCCACGCCCTCTGCTCCCGGCTAGAAAGTGCGCTGCAGGACACTGTGAAGGAGCAAGACAGAAGCTTCACGACTCTAGACTGGAGCTGGTTACAGATGGAGGATGAAGAAAGGTGTAGCCTGGAGCAGGCCTGTGACTGA

**pMX-IKK2kd:**

ATGAGCTGGTCACCGTCCCTCCCAACCCAGACATGTGGAGCCTGGGAAATGAAAGAACGCCTGGGGACCGGGGGATTTGGAAACGTCATCCGGTGGCACAATCAGGCGACAGGTGAACAGATCGCCATCATGCAATGCCGACAGGAGCTCAGCCCAAAGAACAGAGACCGCTGGTGCCTCGAAATCCAGATCATGAGAAGGCTGAACCATCCCAATGTGGTGGCTGCCCGGGATGTCCCAGAGGGGATGCAGAACCTGGCACCCAATGATTTGCCACTGCTGGCCATGGAGTACTGCCAAGGAGGAGATCTCCGAAGATACTTGAACCAGTTCGAGAACTGCTGTGGCCTGCGGGAAGGAGCTGTCCTTACCCTGCTGAGTGACATCGCATCGGCTCTTAGATACCTTCACGAAAACAGAATCATCCATCGAGACCTGAAGCCAGAAAACATCGTTCTGCAGCAAGGAGAGAAAAGATTAATACACAAAATTATTGATCTAGGATATGCCAAGGAGCTGGATCAGGGCGAACTGTGCACGGAATTTGTGGGGACTCTGCAATACCTGGCGCCAGAGCTTCTGGAGCAGCAGAAGTACACCGTGACCGTTGACTACTGGAGCTTCGGCACCCTGGCCTTCGAGTGCATCACTGGCTTCCGGCCCTTCCTCCCTAACTGGCAGCCTGTGCAGTGGCACTCCAAAGTCCGGCAGAAGAGCGAAGTGGACATCGTTGTTAGTGAAGACTTGAATGGAGCAGTGAAGTTTTCAAGTTCGCTACCCTTCCCCAATAATCTTAACAGTGTCTTGGCTGAACGGCTGGAGAAGTGGCTGCAGCTGATGCTTATGTGGCACCCTCGGCAAAGGGGCACGGATCCCCAGTATGGCCCCAACGGCTGCTTCAGAGCCCTGGATGACATCTTGAACTTGAAGCTGGTTCATGTCTTGAACATGGTCACAGGCACCGTTCACACATACCCCGTGACGGAGGATGAGAGTCTGCAGAGCTTAAAAACCAGAATCCAGGAAGACACGGGGATCCTGGAGACAGACCAGGAGCTGCTGCAAGAGGCAGGGCTGGTGCTGCTCCCTGACAAGCCTGCTACTCAGTGCATCTCAGACAGCAAGACAAACGAGGGCCTCACGTTGGACATGGATCTTGTTTTTCTCTTTGACAACAGTAAAATCAACTATGAGACTCAGATCACCCCCCGACCCCAACCGGAAAGTGTCAGCTGTATCCTTCAGGAGCCCAAGCGGAACCTCTCCTTCTTCCAGCTGAGGAAAGTGTGGGGCCAAGTCTGGCACAGCATCCAGACGCTGAAGGAAGACTGTAACCGGCTGCAGCAGGGACAGCGAGCAGCCATGATGAGTCTCCTCCGGAATAACAGCTGCCTCTCTAAGATGAAGAACGCCATGGCCTCCACGGCCCAGCAGCTCAAGGCCAAGCTGGACTTCTTCAAAACCAGCATCCAGATCGACCTGGAGAAGTATAAAGAGCAGACCGAGTTTGGGATCACCTCAGATAAATTGCTGCTGGCTTGGCGGGAGATGGAGCAGGCTGTGGAGCAGTGTGGGCGGGAGAATGACGTGAAGCATCTAGTAGAGCGGATGATGGCACTGCAGACTGACATTGTGGACCTGCAGAGGAGCCCGATGGGTCGGAAGCAGGGGGGCACCCTGGATGACCTAGAGGAACAAGCGAGGGAGCTCTACCGAAGACTCAGGGAGAAGCCAAGAGACCAAAGGACAGAAGGTGACAGCCAGGAGATGGTACGGCTGCTGCTTCAGGCAATCCAAAGCTTTGAGAAGAAAGTTCGGGTGATTTATACACAGCTCAGTAAGACCGTGGTTTGTAAGCAGAAGGCACTGGAGTTGCTGCCCAAGGTAGAAGAGGTAGTGAGCCTTATGAACGAGGACGAGAGGACCGTGGTCCGGCTTCAGGAGAAGCGGCAGAAGGAACTCTGGAACCTCCTGAAGATCGCCTGTAGCAAAGTCCGAGGTCCCGTGAGTGGAAGCCCAGACAGCATGAATGTGTCTCGACTCAGTCACCCTGGTCAGCTAATGTCCCAGCCTTCCAGTGCCTGTGACAGCTTACCTGAATCAGACAAGAAAAGTGAAGAACTGGTGGCCGAAGCCCACGCCCTCTGCTCCCGGCTAGAAAGTGCGCTGCAGGACACTGTGAAGGAGCAAGACAGAAGCTTCACGACTCTAGACTGGAGCTGGTTACAGATGGAGGATGAAGAAAGGTGTAGCCTGGAGCAGGCCTGTGACTGA

**pMX-p65:**

ATGGACGAACTGTTCCCCCTCATCTTCCCGGCAGAGCCAGCCCAGGCCTCTGGCCCCTATGTGGAGATCATTGAGCAGCCCAAGCAGCGGGGCATGCGCTTCCGCTACAAGTGCGAGGGGCGCTCCGCGGGCAGCATCCCAGGCGAGAGGAGCACAGATACCACCAAGACCCACCCCACCATCAAGATCAATGGCTACACAGGACCAGGGACAGTGCGCATCTCCCTGGTCACCAAGGACCCTCCTCACCGGCCTCACCCCCACGAGCTTGTAGGAAAGGACTGCCGGGATGGCTTCTATGAGGCTGAGCTCTGCCCGGACCGCTGCATCCACAGTTTCCAGAACCTGGGAATCCAGTGTGTGAAGAAGCGGGACCTGGAGCAGGCTATCAGTCAGCGCATCCAGACCAACAACAACCCCTTCCAAGTTCCTATAGAAGAGCAGCGTGGGGACTACGACCTGAATGCTGTGCGGCTCTGCTTCCAGGTGACAGTGCGGGACCCATCAGGCAGGCCCCTCCGCCTGCCGCCTGTCCTTTCTCATCCCATCTTTGACAATCGTGCCCCCAACACTGCCGAGCTCAAGATCTGCCGAGTGAACCGAAACTCTGGCAGCTGCCTCGGTGGGGATGAGATCTTCCTACTGTGTGACAAGGTGCAGAAAGAGGACATTGAGGTGTATTTCACGGGACCAGGCTGGGAGGCCCGAGGCTCCTTTTCGCAAGCTGATGTGCACCGACAAGTGGCCATTGTGTTCCGGACCCCTCCCTACGCAGACCCCAGCCTGCAGGCTCCTGTGCGTGTCTCCATGCAGCTGCGGCGGCCTTCCGACCGGGAGCTC

AGTGAGCCCATGGAATTCCAGTACCTGCCAGATACAGACGATCGTCACCGGATTGAGGAGAAACGTAAAAGGACATATGAGACCTTCAAGAGCATCATGAAGAAGAGTCCTTTCAGCGGACCCACCGACCCCCGGCCTCCACCTCGACGCATTGCTGTGCCTTCCCGCAGCTCAGCTTCTGTCCCCAAGCCAGCACCCCAGCCCTATCCCTTTACGTCATCCCTGAGCACCATCAACTATGATGAGTTTCCCACCATGGTGTTTCCTTCTGGGCAGATCAGCCAGGCCTCGGCCTTGGCCCCGGCCCCTCCCCAAGTCCTGCCCCAGGCTCCAGCCCCTGCCCCTGCTCCAGCCATGGTATCAGCTCTGGCCCAGGCCCCAGCCCCTGTCCCAGTCCTAGCCCCAGGCCCTCCTCAGGCTGTGGCCCCACCTGCCCCCAAGCCCACCCAGGCTGGGGAAGGAACGCTGTCAGAGGCCCTGCTGCAGCTGCAGTTTGATGATGAAGACCTGGGGGCCTTGCTTGGCAACAGCACAGACCCAGCTGTGTTCACAGACCTGGCATCCGTCGACAACTCCGAGTTTCAGCAGCTGCTGAACCAGGGCATACCTGTGGCCCCCCACACAACTGAGCCCATGCTGATGGAGTACCCTGAGGCTATAACTCGCCTAGTGACAGGGGCCCAGAGGCCCCCCGACCCAGCTCCTGCTCCACTGGGGGCCCCGGGGCTCCCCAATGGCCTCCTTTCAGGAGATGAAGACTTCTCCTCCATTGCGGACATGGACTTCTCAGCCCTGCTGAGTCAGATCAGCTCCTAA

**pMX-p65SD.**

ATGGACGAACTGTTCCCCCTCATCTTCCCGGCAGAGCCAGCCCAGGCCTCTGGCCCCTATGTGGAGATCATTGAGCAGCCCAAGCAGCGGGGCATGCGCTTCCGCTACAAGTGCGAGGGGCGCTCCGCGGGCAGCATCCCAGGCGAGAGGAGCACAGATACCACCAAGACCCACCCCACCATCAAGATCAATGGCTACACAGGACCAGGGACAGTGCGCATCTCCCTGGTCACCAAGGACCCTCCTCACCGGCCTCACCCCCACGAGCTTGTAGGAAAGGACTGCCGGGATGGCTTCTATGAGGCTGAGCTCTGCCCGGACCGCTGCATCCACAGTTTCCAGAACCTGGGAATCCAGTGTGTGAAGAAGCGGGACCTGGAGCAGGCTATCAGTCAGCGCATCCAGACCAACAACAACCCCTTCCAAGTTCCTATAGAAGAGCAGCGTGGGGACTACGACCTGAATGCTGTGCGGCTCTGCTTCCAGGTGACAGTGCGGGACCCATCAGGCAGGCCCCTCCGCCTGCCGCCTGTCCTTTCTCATCCCATCTTTGACAATCGTGCCCCCAACACTGCCGAGCTCAAGATCTGCCGAGTGAACCGAAACTCTGGCAGCTGCCTCGGTGGGGATGAGATCTTCCTACTGTGTGACAAGGTGCAGAAAGAGGACATTGAGGTGTATTTCACGGGACCAGGCTGGGAGGCCCGAGGCTCCTTTTCGCAAGCTGATGTGCACCGACAAGTGGCCATTGTGTTCCGGACCCCTCCCTACGCAGACCCCAGCCTGCAGGCTCCTGTGCGTGTCTCCATGCAGCTGCGGCGGCCTTCCGACCGGGAGCTC

AGTGAGCCCATGGAATTCCAGTACCTGCCAGATACAGACGATCGTCACCGGATTGAGGAGAAACGTAAAAGGACATATGAGACCTTCAAGAGCATCATGAAGAAGAGTCCTTTCAGCGGACCCACCGACCCCCGGCCTCCACCTCGACGCATTGCTGTGCCTTCCCGCAGCTCAGCTTCTGTCCCCAAGCCAGCACCCCAGCCCTATCCCTTTACGTCATCCCTGAGCACCATCAACTATGATGAGTTTCCCACCATGGTGTTTCCTTCTGGGCAGATCAGCCAGGCCTCGGCCTTGGCCCCGGCCCCTCCCCAAGTCCTGCCCCAGGCTCCAGCCCCTGCCCCTGCTCCAGCCATGGTATCAGCTCTGGCCCAGGCCCCAGCCCCTGTCCCAGTCCTAGCCCCAGGCCCTCCTCAGGCTGTGGCCCCACCTGCCCCCAAGCCCACCCAGGCTGGGGAAGGAACGCTGTCAGAGGCCCTGCTGCAGCTGCAGTTTGATGATGAAGACCTGGGGGCCTTGCTTGGCAACAGCACAGACCCAGCTGTGTTCACAGACCTGGCATCCGTCGACAACTCCGAGTTTCAGCAGCTGCTGAACCAGGGCATACCTGTGGCCCCCCACACAACTGAGCCCATGCTGATGGAGTACCCTGAGGCTATAACTCGCCTAGTGACAGGGGCCCAGAGGCCCCCCGACCCAGCTCCTGCTCCACTGGGGGCCCCGGGGCTCCCCAATGGCCTCCTTTCAGGAGATGAAGACTTCTCCGACATTGCGGACATGGACTTCTCAGCCCTGCTGAGTCAGATCAGCTCCTAA
