# Supplementary material for: Inflammation driven by NF-ĸB compromises bone formation by inducing the differentiation of osteoblasts into atypical inflammatory osteocyte-like cells
Source: Bone Res. 2026 Aug 4;14:80. doi: 10.1038/s41413-026-00546-0 (PMC13439236; doi:10.1038/s41413-026-00546-0)
Supplement: Supplementary file 2 [file 41413_2026_546_MOESM2_ESM.docx]

| ***Gene Name*** | **Primer Sequence** |
| --- | --- |
| *Actb: F* | CTAAGGCCAACCGTGAAAAG |
| *Actb: R* | ACCAGAGGCATACAGGGAC |
| *Alp: F* | GTTGCCAAGCTGGGAAGAACAC |
| *Alp:R* | CCCACCCCGCTATTCCAAAC |
| *Bglap: F* | AAGCAGGAGGGCAATAAGGT |
| *Bglap: R* | TTTGTAGGCGGTCTTCAAGC |
| *Col1: F* | CATGTTCAGCTTTGTGGACCT |
| *Col1: R* | GCAGCTGACTTCAGGGATGT |
| *Dkk1: F* | AGCCATTTTCCTCGAGGGGAA |
| *Dkk1: R* | CAGACGGAGCCTTCTTGTCCTT |
| *Fgf23: F* | TATGGATCTCCACGGCAAC |
| *Fgf23: R* | GTCCACTGGCGGAACTTG |
| *Glut1: F* | TCTCTGTCGGCCTCTTTGTT |
| *Glut1: R* | GCAGAAGGGCAACAGGATAC |
| *Hk2: F* | GATTGTCCGTAACATTCTCATCGA |
| *Hk2: R* | TGTCTTGAGCCGCTCTGAGAT |
| *Ikbkb: F* | CCGGAAAGTGTCAGCTGTATC |
| *Ikbkb: R* | CCTCAGCTGGAAGAAGGAGA |
| *Il1a: F* | GAAGAAGAGACGGCTGAGTTT |
| *Il1a: R* | TCACTCTGGTAGGTGTAAGGT |
| *Il6: F* | GCTACCAAACTGGATATAATCAGGA |
| *Il6: R* | CCAGGTAGCTATGGTACTCCAGAA |
| *Lcn2: F* | CCATCTATGAGCTACAAGAGAACAAT |
| *Lcn2: R* | TCTGATCCAGTAGCGACAGC |
| *Ldha: F* | TGGAAGACAAACTCAAGGGCGAGA |
| *Ldha: R* | TGACCAGCTTGGAGTTCGCAGTTA |
| *Ocn: F* | CAGCGGCCCTGAGTCTGA |
| *Ocn: R* | GCCGGAGTCTGTTCACTACCTTA |
| *Opg: F* | GTTTCCCGAGGACCACAAT |
| *Opg: F* | CCATTCAATGATGTCCAGGAG |
| *Pdpn: F* | CAGTGTTGTTCTGGGTTTTGG |
| *Pdpn: R* | ACCTGGGGTCACAATATCATCT |
| *Rankl: F* | TGAAGACACACTACCTGACTCCTG |
| *Rankl: R* | CCCACAATGTGTTGCAGTTC |
| *Runx:F* | CCCGGGAACCAAGAAATC |
| *Runx:R* | CAGATAGGAGGGGTAAGACTGG |
| *Sost: F* | CCAGGGCTTGGAGAGTACC |
| *Sost: R* | GCAGCTGTACTCGGACACATC |
| *Sp7: F* | CTCTCCTGCAGGCAGTCCT |
| *Sp7: R* | GGGAAGGGTGGGTAGTCATT |
| *Tnfa: F* | CTGTAGCCCACGTCGTAGC |
| *Tnfa: R* | TTGAGATCCATGCCGTTG |

**Table S1: List of qPCR primers.**
