# Supplementary material for: Inflammation driven by NF-ĸB compromises bone formation by inducing the differentiation of osteoblasts into atypical inflammatory osteocyte-like cells
Source: Bone Res. 2026 Aug 4;14:80. doi: 10.1038/s41413-026-00546-0 (PMC13439236; doi:10.1038/s41413-026-00546-0)
Supplement: Supplementary file 3 [file 41413_2026_546_MOESM3_ESM.docx]

Supplemental Figures:

Fig S1. Histological analysis (A)Von-Kossa staining and (B-C) TRAP staining and quantification showing N.Oc/B.Pm in the 7 weeks old WT and SP7-KI mice.

Fig S2. NF-κB activation induces differentiation of OB to aiOCy-L cells. cOB expressing p65, p65-SD (constitutive active phospho mimetic form of p65) and p65-SA (inactive p65) were cultured in osteogenic media (OGM: α-MEM supplemented with 10% FBS, 10 mM β-glycerophosphate, 50 µg/mL of ascorbic acid, and 1% penicillin/streptomycin) for 6 days. cOB^p65^ and cOB^p65SD^ expression results in decreased expression of (A) *Ocn*, (B) *Col1*, (C) *Runx2* and increases expression of (D) *Dkk1*, (E) *Fgf23*, (F) *Pdpn* and (G) *Tnfsf11* or *Rankl*. Immunostaining showing increased E11 expression in cOB^p65^ and cOB^p65SD^ compared to cOB^GFP^ and p65^SA^. Data show mean ± S.D. *p < 0.05, **p < 0.01 and ***p < 0.001, (one-way ANOVA for 3 groups). Data is representative of three independent experiments.

Fig S3. NF-κB activation induces glycolysis, which regulates accelerated OB to aiOCy-L cells transition. cOB^GFP^, cOB^IKK2ca^ and cOB^IKK2kd^ were cultured in OGM for 6 days. Media was changes on 3^rd^ day. (A) photomicrograph depicting increased acidification of cOB^IKK2ca^ supernatant media compared to cOB^GFP^ and cOB^IKK2kd^. cOB expressing p65, p65-SD (constitutive active phospho mimetic form of p65) were cultured in osteogenic media for 6 days. cOB^p65^ and cOB^p65SD^ expression results in significant increase in expression of glycolytic genes (B) *Glut1*, (C) *Hk2* and (D) *Ldha*. cOB^GFP^, cOB^IKK2ca^ and cOB^IKK2kd^ were cultured for 6 days in OGM with or without 3BP (Hk2 inhbitor:100uM) and FX11 (Ldha inhibitor: 10uM). (E) photomicrograph showing cellular morphology in cOB^GFP^, cOB^IKK2ca^ and cOB^IKK2kd^ with or without 3BP and FX11 (compare cOB^GFP^ control conditions with cOB^IKK2ca^ treated with or without 3BP and FX11). qPCR analysis showed no effect of inhibitors on the expression of OB genes (F) *Alp*, (G) *Col1* (H) *Opg* and (I) *Runx2* in cOB^IKK2ca^. had no significant difference in OB genes the expression of OCy gene. Data show mean ± S.D. *p < 0.05, **p < 0.01 and ***p < 0.001, (one-way ANOVA for 3 groups, two way-ANOVA for multiple groups with more than one variable). Data is representative of three independent experiments.

Fig S4. mTOR signaling regulates OB to aiOCy-L cells transition under inflammation. WT cOB were treated with TNFα (10ng/ml) for different time points.(A) WB showed increased expression of pAKTS473/total-AKT. cOB^GFP^, cOB^IKK2ca^ and cOB^IKK2kd^ were cultured in osteogenic media with or without mTOR1/2 inhibitor PP242 (and/or rapamycin) at 1uM concentration for 6 days. qPCR analysis showed that mTOR inhibitor does not decrease expression of (C) *Col1* and (D) *Runx2* in cOB^IKK2ca^ cells. (E) photomicrograph showing cellular morphology in cOB^GFP^, cOB^IKK2ca^ and cOB^IKK2kd^ with or without PP242 and rapamycin (compare cOB^GFP^ control conditions with cOB^IKK2ca^ treated with or without PP242 and Rapamycin). Data show mean± S.D. ****p < 0.0001, (two way-ANOVA for multiple groups with more than one variable). Data is representative of three independent experiments.
